# Supplementary material for: Effect of Baduanjin exercises on elevated blood lipid levels of middle-aged and elderly individuals: protocol for a systematic review and meta-analysis of randomised controlled trials
Source: BMJ Open. 2017 Sep 3;7(9):e017213. doi: 10.1136/bmjopen-2017-017213 (PMC5589011; doi:10.1136/bmjopen-2017-017213)
Supplement: Author's manuscript [file bmjopen-2017-017213.draft_revisions.pdf]

BMJ Open is committed to open peer review. As part of this commitment we make the peer review history of every article we publish publicly available.

When an article is published we post the peer reviewers' comments and the authors' responses online. We also post the versions of the paper that were used during peer review. These are the versions that the peer review comments apply to.

The versions of the paper that follow are the versions that were submitted during the peer review process. They are not the versions of record or the final published versions. They should not be cited or distributed as the published version of this manuscript.

BMJ Open is an open access journal and the full, final, typeset and author-corrected version of record of the manuscript is available on our site with no access controls, subscription charges or pay-per-view fees (http://bmjopen.bmj.com).

If you have any questions on BMJ Open's open peer review process please email editorial.bmjopen@bmj.com

BMJ Open

Baduanjin exercises for middle-aged and elderly population with blood lipid levels：Protocol for a systematic review of randomised controlled trials

Journal:	*BMJ Open*
Manuscript ID	bmjopen-2017-017213
Article Type:	Protocol
Date Submitted by the Author:	07-Apr-2017
Complete List of Authors:	Wen, Junmao<br>Lin, Tong; Guangzhou university of Chinese medicine<br>Jiang, Chenguang; Guangzhou university of Chinese medicine<br>Peng, Rui; The first affiliated hospital of Guangzhou university of Chinese medicine<br>Wu, Wei
**Primary Subject Heading**:	Complementary medicine
Secondary Subject Heading:	Cardiovascular medicine
Keywords:	Baduanjin exercises, blood lipid levels, Protocol

SCHOLARONE™
Manuscripts

For peer review only

Baduanjin exercises for middle-aged and elderly population with blood lipid levels: Protocol for a systematic review of randomised controlled trials

Authors

Junmao Wen[1] ,Tong Lin[2] , Chenguang Jiang[3] , Rui Peng[4], Wei Wu[5*]

1 Guangzhou university of Chinese medicine, Guangzhou, 510405, China. 515301800@qq.com.

2 Guangzhou university of Chinese medicine, Guangzhou, 510405, China. 929023709@qq.com.

3 Guangzhou university of Chinese medicine, Guangzhou, 510405, China. 770984196@qq.com.

4 The first affiliated hospital of Guangzhou university of Chinese medicine,Guangzhou, 510405, China.1280999540@qq.com.

5 The first affiliated hospital of Guangzhou university of Chinese medicine,Guangzhou, 510405, China.3332564768@qq.com.

Corresponding author

Professor Wei Wu；E-mail: 3332564768@qq.com.

Tel: +8613570211913.

Present address

For peer review only - http://bmjopen.bmj.com/site/about/guidelines.xhtml

Guangzhou university of Chinese medicine, No.12, Ji Chang Road ,Baiyun District, Guangzhou,Guangdong Province,China.

Abstract

Objective: To assess the efficacy and safety of Baduanjin exercises for middle-aged and elderly population with blood lipid levels.

Methods: A systematic literature search would be conducted in 6 databases until March 2017: PubMed, EMBASE, Cochrane Library, Chinese National Knowledge Infrastructure (CNKI), Chinese Science and Technology Periodical Database(VIP), and Wan-fang Database. Randomized controlled trials (RCTs) of Baduanjin exercise for blood lipid levels between middle-aged and elderly people will be included. The primary outcome measures as follows: triglyceride (TC), triglyceride (TG), low-density lipoprotein-cholesterol(LDL-C), high-density lipoprotein-cholesterol(HDL-C). Stata 13.0. software will be used for assessment of risk of bias, data synthesis, sensitivity analysis, meta-regression and subgroup analysis. A funnel plot will be developed to evaluate reporting bias and Begg and Egger tests will be used to assess symmetries of funnel plot. We will conduct the GRADE system to assess the quality of evidence.

For peer review only - http://bmjopen.bmj.com/site/about/guidelines.xhtml

Ethics and dissemination: This systematic review will be published in a peer-reviewed journal. Our result will provide an evidence of the efficacy and safety of Baduanjin exercises for middle-aged and elderly population with blood lipid levels. Trial registration number: PROSPERO CRD 42017060613.

Strengths and limitations of this study

This study will assess the efficacy and safety of Baduanjin exercises for middle-aged and elderly population with blood lipid levels and will provide further clinical evidence.

Two reviewers will conduct the data extraction and assessment of the risk of bias independently.

The GRADE system will be used to further evaluate the assessment of results.

There may be language bias due to included studies in English and Chinese.

There may be clinical heterogeneity derived from the frequency, duration of treatment and other variations in the foot-bath with herbal medicine of included studies.

Introduction

Description of the condition

For peer review only - http://bmjopen.bmj.com/site/about/guidelines.xhtml

Elevated blood lipid levels are risk factors leading to diseases such as cardiovascular diseases, stroke, diabetes and so on.[12] Abnormal blood lipid metabolism characterized by the increase of low density lipoprotein -cholesterol (LDL-C) or triglyceride (TC) is a critical factor generating atherosclerotic cardiovascular disease (ASCVD).[34] Currently, statins, fibrates, nicotinic acid and bile acid sequestrants are proved effective in regulating blood lipid.[56] Statins are the most widely used pharmacologic agents, which significantly lower the level of TC and LDL-C that reduce cardiovascular events. The role of statins in primary prevention of high-risk population of cardiovascular diseases has been recognized.[7] However, statins have adverse effects on muscles including myosalgia, initis and rhabdomyolysis.[8] Furthermore, the long term use of statins at large doses can induce liver dysfunction.[9] Fortunately, the traditional cultivation health methods, such as Tai Chi, yoga, Baduanjin, are popular with middle-aged and old people for the reason that exercises are more conducive to cultivate body and promote health.[1011]

Description of the intervention

Baduanjin, originated over 1000 years ago from China, is a qigong comprising of eight sections of gentle movements as well as relaxing postures and integrating into the traditional Chinese medicine theory of Qi.[12] Compared with other exercise therapy, Baduanjin is not complicated

For peer review only - http://bmjopen.bmj.com/site/about/guidelines.xhtml

to master in a short time and demands less on physical qualities.[13] Baduanjin exercises can help stretch out limbs and muscles and regulate the breath, which improves the coordination of body, harmonizes qi and blood, and contributes to physical and mental comfort at the same time.[14] Baduanjin exercises have been generalized between middle-aged and elderly population owing to the fact that they can maintain body and mind in a good state after practicing a period of time.[1516]

Data came from many clinical trials reveal that Baduanjin exercises produce a great effect on lowering the level of TC, triglyceride (TG), LDL-C and increasing the high-density lipoprotein-cholesterol (HDL-C) level, while some other reports indicate that there are no significant differences for effects of blood lipid by practicing Baduanjin.[17] However, relevant systematic review and meta-analysis on evaluating Baduanjin exercises' role in blood lipid for middle-aged and elderly population haven't been reported. In addition, evidence is insufficient to support the widespread use of Baduanjin. Consequently, it's worthwhile to explore efficient exercise therapy beyond current pharmacological therapy and make an assessment on its efficacy on blood lipid. In this study, we conduct a systematic review and meta-analysis to evaluate current evidence concerning effects of Baduanjin for blood lipid in middle-aged and old participants.

For peer review only - http://bmjopen.bmj.com/site/about/guidelines.xhtml

METHODS AND ANALYSIS

Eligibility criteria

Types of Studies

Randomized controlled trials (RCTs) for evaluating the efficacy and safety of Baduanjin exercise in blood lipid between middle-aged and elderly people will be included regardless of the length of treatment.

Types of participants

Participants of middle-aged and elderly, including physically healthy population, patients diagnosed with hypertension based on 2010 Chinese guidelines for the management of hypertension[18] or the definition of diabetes should accord with the World Health Organization[19] will be included.

Types of intervention

Patients of control group includes no treatment, routine treatment or other exercise therapy like jogging or walking based on Conventional therapy. The intervention for treatment group are taking Baduanjin exercises regardless of duration and frequency Conventional therapy.

Types of outcome measures.

The primary outcome measures should include four blood lipid indexes,

For peer review only - http://bmjopen.bmj.com/site/about/guidelines.xhtml

TC, TG, LDL-C, and HDL-C.

The secondary outcome measures will be the body mass index (BMI), waist to hip ratio (WHR), ApoA1, ApoB and adverse events reported in the studies.

search strategy and identification of studies

We will perform medical retrieval in the following database: PubMed, EMBASE, Cochrane Library, Chinese National Knowledge Infrastructure (CNKI), Wanfang Data Information Site, Chinese BioMedical Database (CBM), Chinese Science and Technique Journals Database(VIP). The publication time is from inception to March of 2017. The search terms are: (‘blood lipid’ OR ‘blood fats’ OR ‘hyperlipidemia’ OR ‘hyperlipaemia’ OR ‘hypercholesterolemia’ OR ‘hypertriglyceridemia’ OR ‘hyperlipoproteinemia’ OR ‘cholesterol’) AND (‘Qigong’ OR ‘Baduanjin’ OR ‘Baduanjin exercise’ OR ‘eight section brocades’). Chinese translations of these terms will be applied to Chinese database. The search strategy for PubMed is given in the Table 1.

Two reviewers (TL and GC) will review the full texts of potential eligible studies independently. Discrepancies for inclusion in the meta-analysis will be discussed and settled by a third reviewer. A Preferred Reporting Items for Systematic Reviews and Meta-Analyses (PRISMA) flow chart will be produced to list articles identified, screened, included and

For peer review only - http://bmjopen.bmj.com/site/about/guidelines.xhtml

excluded in details and ascertain eligible studies. Study selections will be show in a PRISMA flow chart (http://www.prisma-statement.org) (figure 1).

study selection and data extraction

Articles will be excluded if no data on blood lipid outcomes can be extracted, the information are inadequate to acquire, or studies are duplication reporting the same results. According to the the criteria established above, two reviewers (TL and GC) will independently review titles and abstracts to select potential references and then scan all full articles to decide whether they are consistent with our study. Endnote X7 will be used to manage literature and remove duplications.

The reviewers will then extract data regarding details of study population, intervention and outcome measures based on a self developed data extraction form. We will utilize a standardized data extraction form including participants, methods, interventions and outcomes. The data extraction form included items listed as follows, general information, trial characteristics, participants, interventions and outcomes. The disagreement will be resolved through consultation and if necessary, discrepancies will be discussed with a third author.

The solution to missing data or unclear scale type

We will send email or make a telephone call on original authors for

For peer review only - http://bmjopen.bmj.com/site/about/guidelines.xhtml

requiring missing data or unclear evaluation scales. If this way cannot obtain the sufficient information, we will just analyze the available data. We will add the potential impact on review results which can be seen in the discussion section with insufficient data.

Risk of bias in included studies

Risk of bias for each included article will be evaluated on the basis of the Cochrane Handbook for Systematic Reviews of Interventions (Chapter 8.5) (Higgins 2011). This involve seven aspects: random sequence generation, allocation concealment, blinding of outcome assessments, incomplete outcome data, selective outcome reporting and other biases. Bias among participants and investigators won't be considered owing that Baduanjin exercise makes blinding impossible. We will make judgments about these risk-related criteria: low, high, or unclear (indicating unclear or unknown risk of bias).

Data synthesis and analysis

We will analyze the data by the means of Stata 13.0. We will use the weighted mean difference (WMD) as the analysis of statistics. All analysis will be calculated 95% CI. The Q and I^2 test statistics will be used to assess the heterogeneity of included studies. For Q statistics, $p<0.05$ is regarded to have significant difference. As to I^2 statistics, $I^2<$ 25% indicates no significant heterogeneity. I^2= 25-50% is considered

For peer review only - http://bmjopen.bmj.com/site/about/guidelines.xhtml

moderate heterogeneity and strong heterogeneity exists when I^2> 50%. We will apply fixed effects models when there are no heterogeneity among studies. Otherwise, the random effects models will be used.

Additional analyses

We will perform sensitivity analysis, meta-regression and subgroup stratified analysis on the basis of study characteristics such as: types of studies, study location, sample size, study quality, adjusting of confounders or not, Baduanjin regimens, and other relevant parameters, to explore the potential sources of heterogeneity. When data extraction is insufficient, qualitative synthesis will be produced.

Assessment of reporting biases

A funnel plot will be developed to evaluate reporting bias among the included studies. We will use Begg and Egger tests for assessing symmetries of funnel plot and define that $p<0.1$ accounts for the statistical significance on publication bias.

Quality of evidence

Grading of Recommendations Assessment, Development and Evaluation (GRADE) approach will be utilized to provide evidence for quality evaluation of the included studies. Reviewers will take into account limitations of the study, inconsistencies, indirect evidence, inaccuracy,

For peer review only - http://bmjopen.bmj.com/site/about/guidelines.xhtml

publication bias. The quality evidence will be graded as four levels, high, moderate, low or very low.

Ethics and dissemination

This systematic review will be published in a peer-reviewed journal. Our result will provide an evidence of the efficacy and safety of Baduanjin exercise for blood lipid levels between middle-aged and elderly people. This review will not related to ethics due to all data with no concerns regarding privacy.

Discussion

Baduanjin is a qigong which has more than one thousand years of history in China, the typical characteristic of which is slow, relaxing and systematic movements more suitable for physically weaker and elderly patients.[20] While the action is soft and slow, it can promote the body muscles, tendons, ligaments fully relaxed and build up the strength of muscles through physical exercise and mind control.[21] Previous studies have indicated that Baduanjin exercise can obviously improve plasma high-density lipoprotein cholesterol (HDL-C) concentrations, or decrease plasma triglyceride (TC), triglycerides (TG), and LDL-C concentrations of the health people or patients.[13] Study shows that qigong can inhibit cholesterol biosynthetic one or more pathways to affect lipid Quality synthesis, such as the promotion of TG transport and degradation,

For peer review only - http://bmjopen.bmj.com/site/about/guidelines.xhtml

increasing TC scavenging ability and transport ability to reduce acetyl coenzyme A.[22] Study also shows that long-term exercise can activate the body's LPL level which is one of the rate-limiting enzymes of triglyceride decomposition determining the degree of skeletal muscle using fatty acids, in order to accelerate lipid metabolism and improve blood lipid levels.[23]

Therefore，Baduanjin could be used for the treatment of high blood lipid level, but the efficacy of this exercise regimen compared to hypolipidemic drugs is unknown. Besides, current evidence is insufficient to contributing to a conclusion that Baduanjin exercise can lower the abnormal blood lipid level. The basic purpose of this review was to systematically assess the effectiveness of Baduanjin exercise on blood lipid in middle-aged and elderly population. This meta-analysis will employ adequate samples as soon as possible to make it more objective and reasonable. It is expected that Baduanjin exercise will have an positive effect on primary prevention of high blood lipid level in middle-aged and elderly population. In summary, this review will be the first to evaluate the impact of Baduanjin exercise on primary prevention of high blood lipid level in middle-aged and elderly population. The results of this review will help to establish the better approach for preventing blood lipid level in high risk groups and provide reliable evidence for its application.

For peer review only - http://bmjopen.bmj.com/site/about/guidelines.xhtml

Contributors

WW and JW conceived the study and drafted the protocol. RP revised it. TL and CJ developed the search strategies, conducted data collection and analyzed independently. All authors have approved the final manuscript.

Funding

This study is supported by the project of the Administration of Traditional Medicine of Guangdong (grant no. 2015 LP01).

Competing interests None declared.

Provenance and peer review Not commissioned; externally peer reviewed.

Data sharing statement No additional data is available.

REFERENCES

1. Zheng GH CB, Fang QY, et al. Primary prevention for risk factors of ischemic stroke with Baduanjin exercise intervention in the community elder population: study protocol for a randomized controlled trial. Trials 2014;**15**.

2. Posadzki P AA, Khalil, MM, et al. . Complementary and alternative medicine for lowering blood lipid levels: A systematic review of systematic reviews. Complement Ther Med 2016;**29**:141-51.

3. Stone NJ RJ, Lichtenstein AH, et al. 2013 ACC/AHA guideline on the treatment of blood cholesterol to reduce atherosclerotic cardiovascular risk in adults: a report of the American College of Cardiology/American Heart Association Task Force on Practice Guidelines. Journal of the American College of Cardiology 2014;**63**:2889-934.

4. SK Y. We shold strength relationship between blood pressure test and clinical trials. Chinese Journal of Clinical Laboratory Science 2008(04):243-45.

For peer review only - http://bmjopen.bmj.com/site/about/guidelines.xhtml

5. Jun M FC, Lv J,et al. Effects of fibrates on cardiovascular outcomes: a systematic review and meta-analysis. Lancet (London, England) 2010;**375**(9729):1875-84.

6. Jacobson TA IM, Maki KC, et al. National lipid association recommendations for patient-centered management of dyslipidemia: part 1--full report. J Clin Lipidol 2015;**9**(2):129-69.

7. Zhu JR GR, Zhao SP,et al. Guidelines for prevention and treatment of dyslipidemia in Chinese adults (revised 2016). Chinese Circulation Journal 2016;**31**(10):937-53.

8. Rosenson RS BS, Jacobson TA, et al. An assessment by the Statin Muscle Safety Task Force: 2014 update. J Clin Lipidol 2014;**8**(3 Suppl):S58-71.

9. McKenney JM DM, Jacobson TA, et al. Final conclusions and recommendations of the National Lipid Association Statin Safety Assessment Task Force. The American journal of cardiology 2006;**97**(8A):89C-94C.

10. Xiong X WP, Li S, et al. Effect of Baduanjin exercise for hypertension: a systematic review and meta-analysis of randomized controlled trials. Maturitas 2015;**80**(4):370-8.

11. MY. C. The Theory and Practice of Health Cultivation Qigong Exercise in Traditional Chinese Medicine. Hu li za zhi The journal of nursing 2015;**62**(6):13-9.

12. Chen HH YM, Lee FY, et al. The effects of Baduanjin qigong in the prevention of bone loss for middle-aged women. The American journal of Chinese medicine 2006;**34**(5):741-7.

13. Zhou XQ ZY, Yang BL, et al. Effects of health Qigong and Baduanjin on the middle and old-aged people's blood lipid. Journal of Beijing Sport University 2007;**30**(06):795-97.

14. An B DK, Zhu Z, Wang Y, et al. Baduanjin alleviates the symptoms of knee osteoarthritis. Journal of alternative and complementary medicine (New York, NY) 2008;**14**(2):167-74.

15. Li R JL, Hong P, et al. The Effect of Baduanjin on Promoting the Physical Fitness and Health of Adults. Evid Based Complement Alternat Med 2014;**2014**:784059.

16. Li M FQ, Li J, et al. The Effect of Chinese Traditional Exercise-Baduanjin on Physical and Psychological Well-Being of College Students: A Randomized Controlled Trial. PLoS One 2015;**10**(7):e0130544.

17. Mei L CQ, Ge L, et al. Systematic Review of Chinese Traditional Exercise Baduanjin Modulating the Blood Lipid Metabolism. Evid Based Complement Alternat Med 2012;**2012**:282131.

18. LS. L. 2010 Chinese Guidelines for the management of hypertension. Chinese Journal of Hypertension 2010;**19**(8):701-43.

19. K.G.M.M. Alberti PZZftWC. Definition, Diagnosis and Classification of Diabetes Mellitus and its

For peer review only - http://bmjopen.bmj.com/site/about/guidelines.xhtml

Complications Part 1: Diagnosis and Classification of Diabetes Mellitus Provisional Report of a WHO Consultation. Diabete Medicine 1999:539-53.

20. Tsang HW CL, Lak DC. Qigong as a psychosocial intervention for depressed elderly with chronic physical illnesses. International journal of geriatric psychiatry 2002;**17**(12):1146-54.

21. Yan SX SY, Lang JL, et al. The Effect of Baduanjin on Blood Glucose and Blood Lipid Metabolism in male inpatients with Schizophrenia. Guiding Journal of Traditional Chinese Medicine and Pharmacy 2012;**18**(05):116-17.

22. Wang B WZ, Lu ST, et al. Effects of Qigong and Mawangdui Guided exercise on Blood Lipid and Free Radical Metabolism in middle-aged and elderly women. Chinese Journal of Gerontology 2014(13):3720-22.

23. Wooten JS BK, Anderson C. Response of lipid, lipoprotein-cholesterol, and electrophoretic characteristics of lipoproteins following a single bout of aerobic exercise in women. European journal of applied physiology 2008;**104**(1):19-27.

Table 1 Search strategy utilized in the PubMed database

NO	Search terms
1	blood lipid
2	blood fats
3	hyperlipidemia
4	hyperlipaemia
5	hypercholesterolemia
6	hypertriglyceridemia
7	hyperlipoproteinemia
8	cholesterol
9	or1-8

For peer review only

For peer review only - http://bmjopen.bmj.com/site/about/guidelines.xhtml

10	Qigong
11	Baduanjin
12	Baduanjin exercise
13	eight section brocades
14	Or 10-14
15	And 9-14

For peer review only

For peer review only - http://bmjopen.bmj.com/site/about/guidelines.xhtml

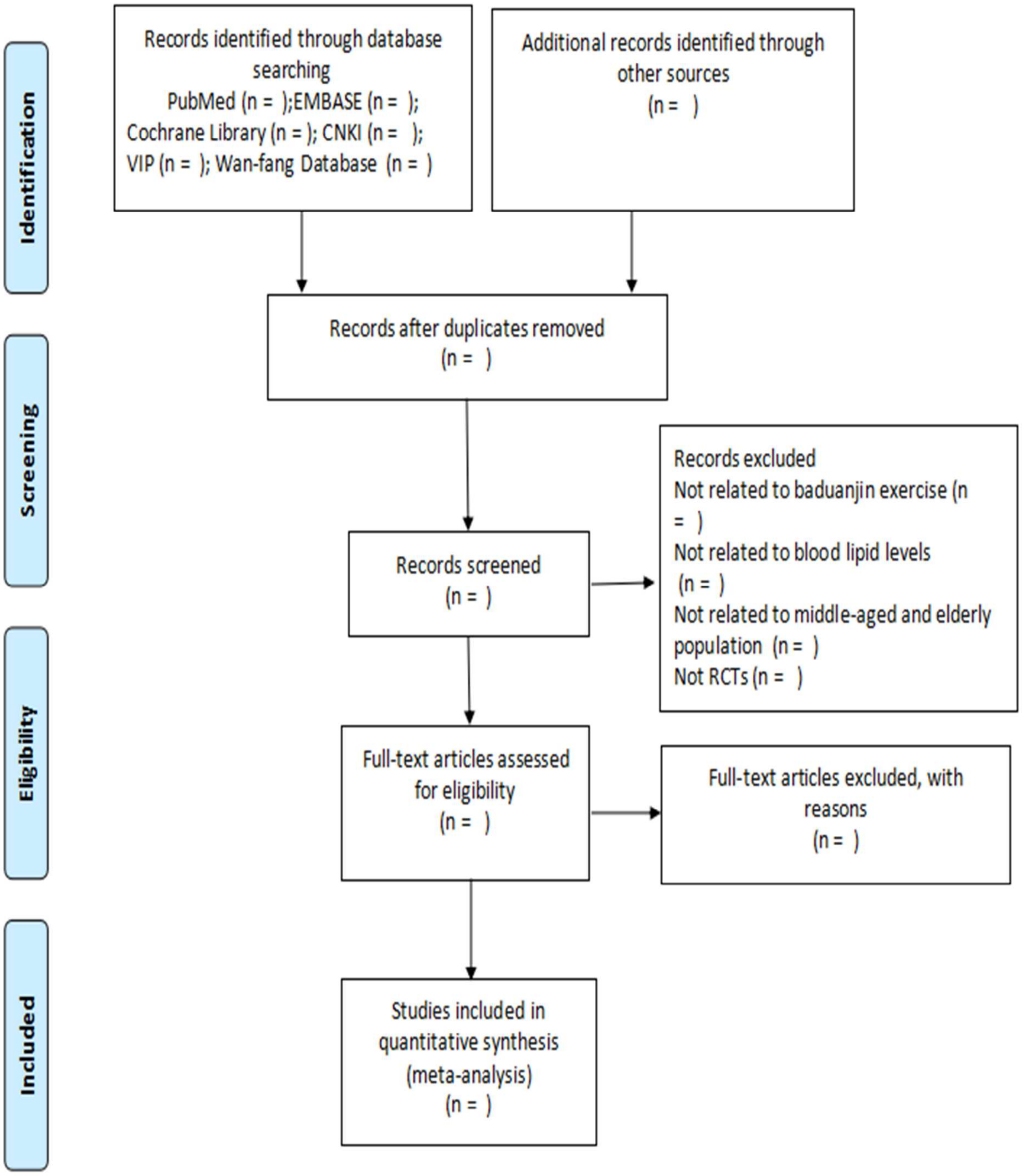

Figure 1 Flow diagram of selection process.

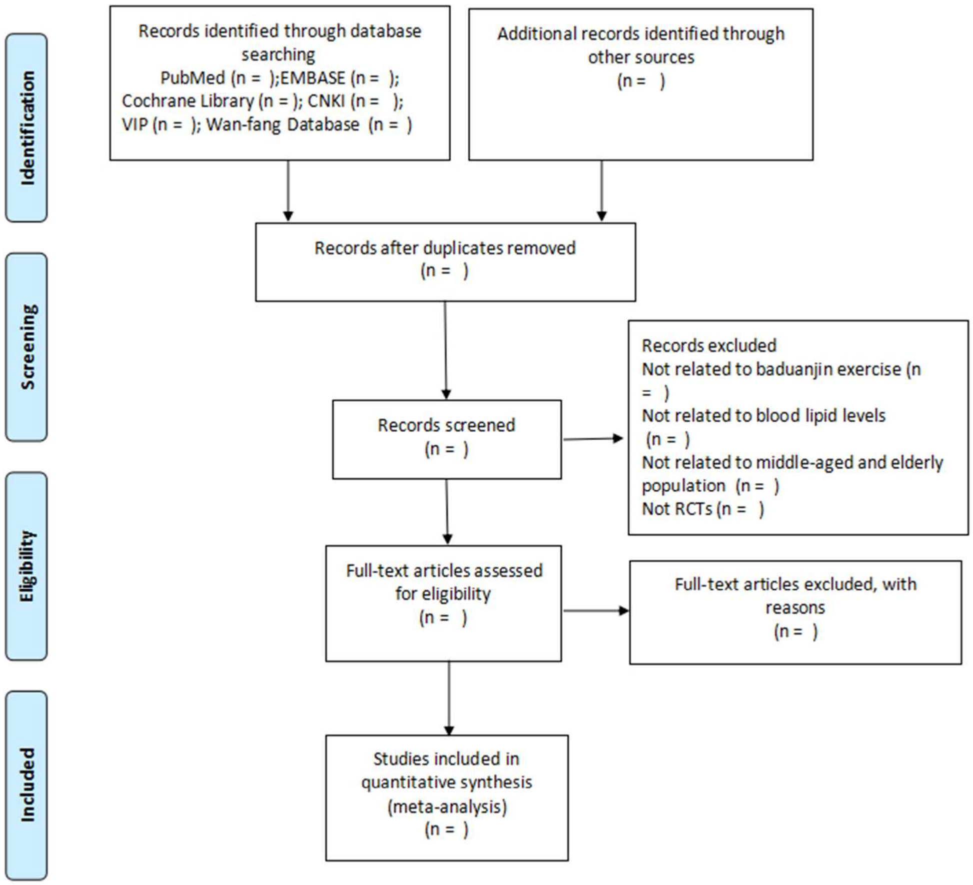

For peer review only - http://bmjopen.bmj.com/site/about/guidelines.xhtml

PRISMA-P (Preferred Reporting Items for Systematic review and Meta-Analysis Protocols) 2015 checklist: recommended items to address in a systematic review protocol*

Section and topic	Item No	Checklist item	Reported on page #
**ADMINISTRATIVE INFORMATION**			
Title:			
Identification	1a	Identify the report as a protocol of a systematic review	1
Update	1b	If the protocol is for an update of a previous systematic review, identify as such	no
Registration	2	If registered, provide the name of the registry (such as PROSPERO) and registration number	2
Authors:			
Contact	3a	Provide name, institutional affiliation, e-mail address of all protocol authors; provide physical mailing address of corresponding author	1
Contributions	3b	Describe contributions of protocol authors and identify the guarantor of the review	11-12
Amendments	4	If the protocol represents an amendment of a previously completed or published protocol, identify as such and list changes; otherwise, state plan for documenting important protocol amendments	no
Support:			
Sources	5a	Indicate sources of financial or other support for the review	12
Sponsor	5b	Provide name for the review funder and/or sponsor	12
Role of sponsor or funder	5c	Describe roles of funder(s), sponsor(s), and/or institution(s), if any, in developing the protocol	12
**INTRODUCTION**			
Rationale	6	Describe the rationale for the review in the context of what is already known	3-5
Objectives	7	Provide an explicit statement of the question(s) the review will address with reference to participants, interventions, comparators, and outcomes (PICO)	1
**METHODS**			
Eligibility criteria	8	Specify the study characteristics (such as PICO, study design, setting, time frame) and report characteristics (such as years considered, language, publication status) to be used as criteria for eligibility for the review	5
Information sources	9	Describe all intended information sources (such as electronic databases, contact with study authors, trial registers or other grey literature sources) with planned dates of coverage	6
Search strategy	10	Present draft of search strategy to be used for at least one electronic database, including planned limits, such that it could be repeated	6,7

For peer review only

Study records:			
Data management	11a	Describe the mechanism(s) that will be used to manage records and data throughout the review	7
Selection process	11b	State the process that will be used for selecting studies (such as two independent reviewers) through each phase of the review (that is, screening, eligibility and inclusion in meta-analysis)	7
Data collection process	11c	Describe planned method of extracting data from reports (such as piloting forms, done independently, in duplicate), any processes for obtaining and confirming data from investigators	7,8
Data items	12	List and define all variables for which data will be sought (such as PICO items, funding sources), any pre-planned data assumptions and simplifications	8
Outcomes and prioritization	13	List and define all outcomes for which data will be sought, including prioritization of main and additional outcomes, with rationale	6
Risk of bias in individual studies	14	Describe anticipated methods for assessing risk of bias of individual studies, including whether this will be done at the outcome or study level, or both; state how this information will be used in data synthesis	8
Data synthesis	15a	Describe criteria under which study data will be quantitatively synthesised	9
	15b	If data are appropriate for quantitative synthesis, describe planned summary measures, methods of handling data and methods of combining data from studies, including any planned exploration of consistency (such as I^2, Kendall's τ)	9
	15c	Describe any proposed additional analyses (such as sensitivity or subgroup analyses, meta-regression)	
	15d	If quantitative synthesis is not appropriate, describe the type of summary planned	9
Meta-bias(es)	16	Specify any planned assessment of meta-bias(es) (such as publication bias across studies, selective reporting within studies)	9
Confidence in cumulative evidence	17	Describe how the strength of the body of evidence will be assessed (such as GRADE)	10

*** It is strongly recommended that this checklist be read in conjunction with the PRISMA-P Explanation and Elaboration (cite when available) for important clarification on the items. Amendments to a review protocol should be tracked and dated. The copyright for PRISMA-P (including checklist) is held by the PRISMA-P Group and is distributed under a Creative Commons Attribution Licence 4.0.**

From: Shamseer L, Moher D, Clarke M, Ghersi D, Liberati A, Petticrew M, Shekelle P, Stewart L, PRISMA-P Group. Preferred reporting items for systematic review and meta-analysis protocols (PRISMA-P) 2015: elaboration and explanation. BMJ. 2015 Jan 2;349(jan02 1):g7647.

For peer review only

BMJ Open

Effect of Baduanjin exercises on elevated blood lipid levels of middle-aged and elderly individuals: Protocol for a systematic review and meta-analysis of randomized controlled trials

Journal:	*BMJ Open*
Manuscript ID	bmjopen-2017-017213.R1
Article Type:	Protocol
Date Submitted by the Author:	22-Jun-2017

Complete List of Authors:	Wen, Junmao<br>Lin, Tong; Guangzhou university of Chinese medicine<br>Jiang, Chenguang; Guangzhou university of Chinese medicine<br>Peng, Rui; The first affiliated hospital of Guangzhou university of Chinese medicine<br>Wu, Wei

**Primary Subject Heading**:	Complementary medicine
Secondary Subject Heading:	Cardiovascular medicine
Keywords:	Baduanjin exercises, blood lipid levels, Protocol

SCHOLARONE™ Manuscripts

For peer review only

Effect of Baduanjin exercises on elevated blood lipid levels of middle-aged and elderly individuals: Protocol for a systematic review and meta-analysis of randomized controlled trials

Authors

Junmao Wen[1], Tong Lin[2], Chenguang Jiang[3], Rui Peng[4], Wei Wu[5*]

[1]Guangzhou University of Chinese Medicine, Guangzhou 510405, China

Email: 515301800@qq.com.

[2]Guangzhou University of Chinese Medicine, Guangzhou 510405, China

Email: 929023709@qq.com.

[3]Guangzhou University of Chinese Medicine, Guangzhou 510405, China

Email: 770984196@qq.com.

[4]The First Affiliated Hospital of Guangzhou University of Chinese Medicine, Guangzhou 510405, China

Email: 1280999540@qq.com.

[5]The First Affiliated Hospital of Guangzhou University of Chinese Medicine, Guangzhou 510405, China

Email: 3332564768@qq.com.

Corresponding author

Professor Wei Wu

Email: 3332564768@qq.com

Tel: +8613570211913

Present address

For peer review only

For peer review only - http://bmjopen.bmj.com/site/about/guidelines.xhtml

Guangzhou University of Chinese Medicine, No. 12, Ji Chang Road, Baiyun District, Guangzhou 510405, China

Abstract

Objective: To assess the safety and effect on elevated blood lipid levels of Baduanjin exercises in middle-aged and elderly individuals.

Methods: A systematic literature search for articles up to March 2017 will be conducted using seven databases: PubMed, EMBASE, Cochrane Library, Chinese National Knowledge Infrastructure, Chinese Science and Technology Periodicals Database, Chinese BioMedical Database, and Wanfang Data. Inclusion criteria are randomized controlled trials of Baduanjin exercises that examine blood lipid levels in middle-aged and elderly individuals. The primary outcome measures will be total cholesterol, triglycerides, low-density lipoprotein cholesterol, and high-density lipoprotein cholesterol. Stata 13.0. software will be used for data synthesis, sensitivity analysis, meta-regression, subgroup analysis, and risk of bias assessment. A funnel plot will be developed to evaluate reporting bias and Begg and Egger tests will be used to assess funnel plot symmetries. We will use the Grading of Recommendations Assessment, Development and Evaluation system to assess the quality of evidence.

Ethics and dissemination: This systematic review will be submitted to a peer-reviewed journal. Our findings will provide information about the

For peer review only

For peer review only - http://bmjopen.bmj.com/site/about/guidelines.xhtml

safety of Baduanjin exercises for middle-aged and elderly individuals and their effect on elevated blood lipid levels. Trial registration number: PROSPERO CRD 42017060613.

Strengths and limitations of this study

- This study will assess the safety of Baduanjin exercises and their effect on elevated blood lipid levels in middle-aged and elderly individuals.
- Two reviewers will independently conduct the data extraction and risk of bias assessment.
- The Grading of Recommendations Assessment, Development and Evaluation system will be used to further evaluate study findings.
- There may be a language bias, as both English and Chinese studies will be included.
- There may be clinical heterogeneity because of variations in treatment frequency and duration and the use of additional therapies (e.g., herbal medicine).

INTRODUCTION

Description of the condition

Elevated blood lipid levels are risk factors for problems such as cardiovascular disease, stroke, and diabetes.[12] Abnormal blood lipid metabolism, characterized by an increase in low-density lipoprotein cholesterol (LDL-C) or total cholesterol (TC), is a critical factor for

For peer review only - http://bmjopen.bmj.com/site/about/guidelines.xhtml

subsequent atherosclerotic cardiovascular disease.[34] Statins, fibrates, nicotinic acid, and bile acid sequestrants are effective in regulating blood lipids.[56] Statins are the most widely used pharmacologic agents. They significantly lower TC and LDL-C levels, which reduces cardiovascular events. The role of statins in the primary prevention of cardiovascular diseases in high-risk populations has been recognized.[7] However, statins have adverse effects on muscles, leading to problems such as myosalgia, initis, and rhabdomyolysis.[8] Furthermore, the long-term use of high doses of statins can induce liver dysfunction.[9] Fortunately, traditional cultivation health methods, such as Tai Chi, yoga, and Baduanjin, are popular with middle-aged and older people and are considered to cultivate the body and promote health.[1011]

Description of the intervention

Baduanjin, which originated in China over 1000 years ago, is a qigong practice comprising eight sections of gentle movements and relaxation postures. It is based on the traditional Chinese medical theory of qi.[12] Compared with other exercise therapies, Baduanjin is easy to master in a short time and has fewer physical demands.[13] Baduanjin exercises can help stretch limbs and muscles and regulate the breath, which improves the coordination of the body, harmonizes qi and blood, and contributes to physical and mental well-being.[14] Baduanjin exercises are popular in the middle-aged and elderly population because regular practice helps

For peer review only - http://bmjopen.bmj.com/site/about/guidelines.xhtml

maintain positive physical and mental states.[15,16]

Many clinical trials have shown that Baduanjin exercises can substantially lower levels of TC, triglyceride (TG), and LDL-C and can increase high-density lipoprotein cholesterol (HDL-C) levels. However, other studies have found that Baduanjin practice has no significant effects on blood lipid levels.[17] There have been no relevant systematic reviews and meta-analyses on the effects of Baduanjin on elevated blood lipid levels in middle-aged and elderly individuals. In addition, there is insufficient evidence to support the widespread use of Baduanjin. Consequently, an examination of this therapy's effect on elevated blood lipid levels is needed. In this study, we plan to conduct a systematic review and meta-analysis to evaluate current evidence on the effects of Baduanjin on elevated blood lipid levels in middle-aged and elderly participants.

METHODS AND ANALYSIS

Eligibility criteria

Study type

Regardless of the length of treatment, we will include all randomized controlled trials that evaluated the safety of Baduanjin exercises and their effect on elevated blood lipid levels in middle-aged and elderly people .

Participants

We will include studies of middle-aged and elderly participants including

For peer review only - http://bmjopen.bmj.com/site/about/guidelines.xhtml

the following groups: physically healthy individuals, those with hyperlipidemia, cardiovascular diseases, stroke, hyperlipidemia combined with hypertension (based on 2010 Chinese guidelines for the management of hypertension[18]), or hyperlipidemia combined with diabetes (based on the World Health Organization's definition of diabetes[19]).

Interventions

Control group patients will comprise those receiving no treatment, routine treatment, or other conventional exercise therapies such as jogging or walking. The intervention group will receive Baduanjin exercises of various duration and frequency based on routine regimens.

Outcome measures

The primary outcome measures will comprise four blood lipid indexes: TC, TG, LDL-C, and HDL-C.

The secondary outcome measures will be body mass index, waist-to-hip ratio, ApoA1, ApoB, and adverse events.

Search strategy and identification of studies

We will retrieve articles from the following databases: PubMed, EMBASE, Cochrane Library, Chinese National Knowledge Infrastructure, Wanfang Data, Chinese BioMedical Database, and Chinese Science and Technology Periodicals Database. The publication period will be from inception to March 2017. The search terms will be ("blood lipid" OR "blood fats" OR "hyperlipidemia" OR "hyperlipaemia" OR

For peer review only - http://bmjopen.bmj.com/site/about/guidelines.xhtml

For peer review only

"hypercholesterolemia" OR "hypertriglyceridemia" OR "hyperlipoproteinemia" OR "total cholesterol" OR "triglycerides" OR "low-density lipoprotein cholesterol" OR "high-density lipoprotein cholesterol" OR "dyslipidemia") AND ("Qigong" OR "Baduanjin" OR "Baduanjin exercise" OR "eight section brocades"). Chinese translations of these terms will be used for the Chinese databases. Table 1 shows the search strategy for PubMed.

Two reviewers (TL and CJ) will independently review the full texts of potential eligible studies. Discrepancies about inclusion in the meta-analysis will be discussed and settled by a third reviewer. A Preferred Reporting Items for Systematic Reviews and Meta-Analyses (PRISMA) flow chart will be produced to show the number of articles identified, screened, included, and excluded, reasons for exclusion, and to ascertain eligible studies. The study selection process will be described in a PRISMA flow chart (http://www.prisma-statement.org) (Figure 1).

Study selection and data extraction

We will exclude articles for which no data on blood lipid outcomes is presented, relevant information is unavailable, or results are duplicated. Two reviewers (TL and CJ) will use the criteria described above to independently review titles and abstracts to select potential references and then to scan all full articles for eligibility. Endnote X7 will be used to manage literature and remove duplications.

For peer review only - http://bmjopen.bmj.com/site/about/guidelines.xhtml

The reviewers will then extract data about the study population, intervention, and outcome measures using a self-developed data extraction form. The data extraction form includes the following items: general information, trial characteristics, participants, interventions, and outcomes. Disagreements will be resolved through consultation and if necessary, discrepancies will be discussed with a third author.

Addressing missing data or unclear measurement scales

If necessary, we will contact authors of articles by email or telephone to request missing data or additional information about evaluation scales. If sufficient information cannot be obtained in this way, we will analyze the available data. We will take into account the potential impact of insufficient data on the review results (in the Discussion section).

Risk of bias in included studies

Risk of bias for each included article will be evaluated according to the Cochrane Handbook for Systematic Reviews of Interventions. This recommends the assessment of several sources of bias, including random sequence generation, allocation concealment, blinding of outcome assessments, incomplete outcome data, and selective outcome reporting. Bias among participants and investigators will not be considered because the Baduanjin exercises make blinding impossible. We will judge the risks as low, high, or unclear (unclear or unknown risk of bias).

Data synthesis and analysis

For peer review only - http://bmjopen.bmj.com/site/about/guidelines.xhtml

We will analyze the data using Stata 13.0. We will calculate weighted mean differences and 95% confidence intervals. The Q and I^2 test statistics will be used to assess the heterogeneity of included studies. For the Q statistic, $p < 0.05$ will be considered as indicating significant differences. For the I^2 statistic, $I^2 < 25\%$ indicates no significant heterogeneity, $I^2 = 25–50\%$ is considered moderate heterogeneity, and $I^2 > 50\%$ indicates strong heterogeneity. We will use fixed effects models if there is no heterogeneity among studies and random effects models if there is heterogeneity.

Additional analyses

We will perform sensitivity analysis, meta-regression, and subgroup-stratified analysis based on various study characteristics, such as study type, study location, sample size, study quality, adjustment (or not) for confounders, Baduanjin regimens, and other relevant parameters (e.g., diet and lifestyle) to explore potential sources of heterogeneity. If data extraction is insufficient, we will create a qualitative synthesis.

Assessment of reporting biases

A funnel plot will be developed to evaluate reporting bias of the included studies. We will use Begg and Egger tests to assess funnel plot symmetry and will interpret values of $p < 0.1$ as showing statistical significance (i.e., publication bias).

Quality of evidence

For peer review only - http://bmjopen.bmj.com/site/about/guidelines.xhtml

The Grading of Recommendations Assessment, Development and Evaluation (GRADE) approach will be used to evaluate the quality of evidence of the included studies. Reviewers will take into account limitations of the study, inconsistencies, indirect evidence, inaccuracies, and publication bias. Four levels of evidence quality will be used: high, moderate, low, or very low.

Ethics and dissemination

Our aim is to publish this systematic review in a peer-reviewed journal. Our findings will provide information about the safety of Baduanjin exercises and their effect on blood lipid levels of middle-aged and elderly people. This review will not require ethical approval as there are no issues about participant privacy.

Discussion

Baduanjin is a form of qigong that has been practiced for more than 1000 years in China. Baduanjin comprises slow, relaxing, and systematic movements that are suitable for physically weak and elderly patients.[20] Although the movements are soft and slow, the physical and mental aspects of this practice can strengthen the body muscles and tendons and relax the ligaments.[21] Previous studies have indicated that Baduanjin can obviously increase plasma HDL-C concentrations and decrease plasma TC, TG, and LDL-C concentrations in both healthy people and patients.[13] One study showed that qigong can inhibit cholesterol biosynthesis in one

For peer review only - http://bmjopen.bmj.com/site/about/guidelines.xhtml

or more pathways to improve lipid synthesis (e.g., by promoting TG transport and degradation, increasing TC scavenging ability, and increasing TC ability to transport and reduce acetyl coenzyme A).[22] Research has also shown that long-term exercise can activate levels of lipoprotein (a rate-limiting enzyme that causes TG decomposition and determines the amount of fatty acids in skeletal muscle), which accelerates lipid metabolism and improves blood lipid levels.[23]

Baduanjin could therefore be used to treat high blood lipid levels, but the efficacy of this exercise regimen compared with hypolipidemic drugs is unknown. In addition, there is insufficient evidence that Baduanjin exercise can lower abnormal blood lipid levels. The purpose of this review is to systematically assess the effect of Baduanjin exercises on blood lipid levels in middle-aged and elderly individuals. We aim to use enough studies to ensure adequate power for the meta-analysis. We expect to find that Baduanjin exercises have a positive effect on primary prevention of high blood lipid levels in middle-aged and elderly individuals. In summary, this review will be the first to evaluate the impact of Baduanjin exercise on primary prevention of high blood lipid levels in middle-aged and elderly individuals. The results of this review may help to establish a better approach to prevention of high blood lipid levels in high-risk groups and to provide reliable evidence for its application.

For peer review only - http://bmjopen.bmj.com/site/about/guidelines.xhtml

REFERENCES

1. Zheng GH CB, Fang QY, et al. Primary prevention for risk factors of ischemic stroke with Baduanjin exercise intervention in the community elder population: study protocol for a randomized controlled trial. Trials 2014;**15**.

2. Posadzki P AA, Khalil, MM, et al. . Complementary and alternative medicine for lowering blood lipid levels: A systematic review of systematic reviews. Complement Ther Med 2016;**29**:141-51.

3. Stone NJ RJ, Lichtenstein AH, et al. 2013 ACC/AHA guideline on the treatment of blood cholesterol to reduce atherosclerotic cardiovascular risk in adults: a report of the American College of Cardiology/American Heart Association Task Force on Practice Guidelines. Journal of the American College of Cardiology 2014;**63**:2889-934.

4. SK Y. We shold strength relationship between blood pressure test and clinical trials. Chinese Journal of Clinical Laboratory Science 2008(04):243-45.

5. Jun M FC, Lv J,et al. Effects of fibrates on cardiovascular outcomes: a systematic review and meta-analysis. Lancet (London, England) 2010;**375**(9729):1875-84.

6. Jacobson TA IM, Maki KC, et al. National lipid association recommendations for patient-centered management of dyslipidemia: part 1--full report. J Clin Lipidol 2015;**9**(2):129-69.

7. Zhu JR GR, Zhao SP,et al. Guidelines for prevention and treatment of dyslipidemia in Chinese adults (revised 2016). Chinese Circulation Journal 2016;**31**(10):937-53.

8. Rosenson RS BS, Jacobson TA, et al. An assessment by the Statin Muscle Safety Task Force: 2014 update. J Clin Lipidol 2014;**8**(3 Suppl):S58-71.

9. McKenney JM DM, Jacobson TA, et al. Final conclusions and recommendations of the National Lipid Association Statin Safety Assessment Task Force. The American journal of cardiology 2006;**97**(8A):89C-94C.

10. Xiong X WP, Li S, et al. Effect of Baduanjin exercise for hypertension: a systematic review and meta-analysis of randomized controlled trials. Maturitas 2015;**80**(4):370-8.

11. MY. C. The Theory and Practice of Health Cultivation Qigong Exercise in Traditional Chinese Medicine. Hu li za zhi The journal of nursing 2015;**62**(6):13-9.

12. Chen HH YM, Lee FY, et al. The effects of Baduanjin qigong in the prevention of bone loss for middle-aged women. The American journal of Chinese medicine 2006;**34**(5):741-7.

13. Zhou XQ ZY, Yang BL, et al. Effects of health Qigong and Baduanjin on the middle and old-aged people's blood lipid. Journal of Beijing Sport University 2007;**30**(06):795-97.

14. An B DK, Zhu Z, Wang Y, et al. Baduanjin alleviates the symptoms of knee osteoarthritis. Journal of alternative and complementary medicine (New York, NY) 2008;**14**(2):167-74.

15. Li R JL, Hong P, et al. The Effect of Baduanjin on Promoting the Physical Fitness and Health of Adults. Evid Based Complement Alternat Med 2014;**2014**:784059.

16. Li M FQ, Li J, et al. The Effect of Chinese Traditional Exercise-Baduanjin on Physical and Psychological Well-Being of College Students: A Randomized Controlled Trial. PLoS One 2015;**10**(7):e0130544.

17. Mei L CQ, Ge L, et al. Systematic Review of Chinese Traditional Exercise Baduanjin Modulating the Blood Lipid Metabolism. Evid Based Complement Alternat Med 2012;**2012**:282131.

18. LS. L. 2010 Chinese Guidelines for the management of hypertension. Chinese Journal of Hypertension 2010;**19**(8):701-43.

19. K.G.M.M. Alberti PZZftWC. Definition, Diagnosis and Classification of Diabetes Mellitus and its

For peer review only - http://bmjopen.bmj.com/site/about/guidelines.xhtml

Complications Part 1: Diagnosis and Classification of Diabetes Mellitus Provisional Report of a WHO Consultation. Diabete Medicine 1999:539-53.

20. Tsang HW CL, Lak DC. Qigong as a psychosocial intervention for depressed elderly with chronic physical illnesses. International journal of geriatric psychiatry 2002;**17**(12):1146-54.

21. Yan SX SY, Lang JL, et al. The Effect of Baduanjin on Blood Glucose and Blood Lipid Metabolism in male inpatients with Schizophrenia. Guiding Journal of Traditional Chinese Medicine and Pharmacy 2012;**18**(05):116-17.

22. Wang B WZ, Lu ST, et al. Effects of Qigong and Mawangdui Guided exercise on Blood Lipid and Free Radical Metabolism in middle-aged and elderly women. Chinese Journal of Gerontology 2014(13):3720-22.

23. Wooten JS BK, Anderson C. Response of lipid, lipoprotein-cholesterol, and electrophoretic characteristics of lipoproteins following a single bout of aerobic exercise in women. European journal of applied physiology 2008;**104**(1):19-27.

Authors' contributions

WW and JW conceived the study and drafted the protocol. RP revised the study. TL and CJ developed the search strategies, collected the data, and independently analyzed the data. All authors have approved the final manuscript.

Funding

This study is supported by a project of the Administration of Traditional Medicine of Guangdong (grant no. 2015 LP01).

Competing interests

None declared.

Provenance and peer review Not commissioned; externally peer reviewed.

Data sharing statement

No additional data are available.

For peer review only - http://bmjopen.bmj.com/site/about/guidelines.xhtml

For peer review only

Table 1 Search strategy for the PubMed database

NO	Search terms
1	blood lipid
2	blood fats
3	hyperlipidemia
4	hyperlipaemia
5	hypercholesterolemia
6	hypertriglyceridemia
7	hyperlipoproteinemia
8	total cholesterol
9	triglycerides
10	low-density lipoprotein cholesterol
11	high-density lipoprotein cholesterol
12	dyslipidemia
13	or1-12
14	Qigong
15	Baduanjin
16	Baduanjin exercise
17	eight section brocades
18	Or 14-18
19	And 13-18

Figure 1 Flow diagram of study selection process

For peer review only

For peer review only - http://bmjopen.bmj.com/site/about/guidelines.xhtml

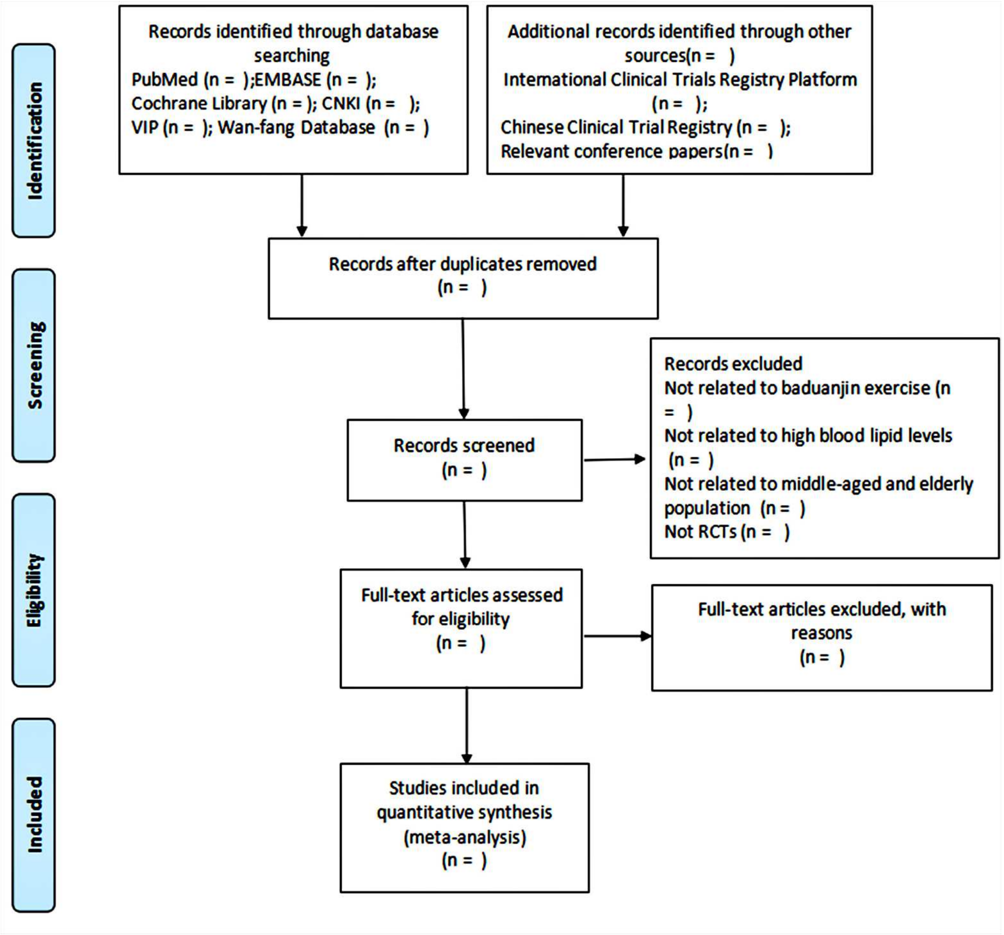

187x175mm (300 x 300 DPI)

PRISMA-P (Preferred Reporting Items for Systematic review and Meta-Analysis Protocols) 2015 checklist: recommended items to address in a systematic review protocol*

Section and topic	Item No	Checklist item	Reported on page #
**ADMINISTRATIVE INFORMATION**			
Title:			
Identification	1a	Identify the report as a protocol of a systematic review	1
Update	1b	If the protocol is for an update of a previous systematic review, identify as such	no
Registration	2	If registered, provide the name of the registry (such as PROSPERO) and registration number	2
Authors:			
Contact	3a	Provide name, institutional affiliation, e-mail address of all protocol authors; provide physical mailing address of corresponding author	1
Contributions	3b	Describe contributions of protocol authors and identify the guarantor of the review	11-12
Amendments	4	If the protocol represents an amendment of a previously completed or published protocol, identify as such and list changes; otherwise, state plan for documenting important protocol amendments	no
Support:			
Sources	5a	Indicate sources of financial or other support for the review	12
Sponsor	5b	Provide name for the review funder and/or sponsor	12
Role of sponsor or funder	5c	Describe roles of funder(s), sponsor(s), and/or institution(s), if any, in developing the protocol	12
**INTRODUCTION**			
Rationale	6	Describe the rationale for the review in the context of what is already known	3-5
Objectives	7	Provide an explicit statement of the question(s) the review will address with reference to participants, interventions, comparators, and outcomes (PICO)	1
**METHODS**			
Eligibility criteria	8	Specify the study characteristics (such as PICO, study design, setting, time frame) and report characteristics (such as years considered, language, publication status) to be used as criteria for eligibility for the review	5
Information sources	9	Describe all intended information sources (such as electronic databases, contact with study authors, trial registers or other grey literature sources) with planned dates of coverage	6
Search strategy	10	Present draft of search strategy to be used for at least one electronic database, including planned limits, such that it could be repeated	6,7

For peer review only

Study records:			
Data management	11a	Describe the mechanism(s) that will be used to manage records and data throughout the review	7
Selection process	11b	State the process that will be used for selecting studies (such as two independent reviewers) through each phase of the review (that is, screening, eligibility and inclusion in meta-analysis)	7
Data collection process	11c	Describe planned method of extracting data from reports (such as piloting forms, done independently, in duplicate), any processes for obtaining and confirming data from investigators	7,8
Data items	12	List and define all variables for which data will be sought (such as PICO items, funding sources), any pre-planned data assumptions and simplifications	8
Outcomes and prioritization	13	List and define all outcomes for which data will be sought, including prioritization of main and additional outcomes, with rationale	6
Risk of bias in individual studies	14	Describe anticipated methods for assessing risk of bias of individual studies, including whether this will be done at the outcome or study level, or both; state how this information will be used in data synthesis	8
Data synthesis	15a	Describe criteria under which study data will be quantitatively synthesised	9
	15b	If data are appropriate for quantitative synthesis, describe planned summary measures, methods of handling data and methods of combining data from studies, including any planned exploration of consistency (such as I^2, Kendall's τ)	9
	15c	Describe any proposed additional analyses (such as sensitivity or subgroup analyses, meta-regression)	
	15d	If quantitative synthesis is not appropriate, describe the type of summary planned	9
Meta-bias(es)	16	Specify any planned assessment of meta-bias(es) (such as publication bias across studies, selective reporting within studies)	9
Confidence in cumulative evidence	17	Describe how the strength of the body of evidence will be assessed (such as GRADE)	10

*** It is strongly recommended that this checklist be read in conjunction with the PRISMA-P Explanation and Elaboration (cite when available) for important clarification on the items. Amendments to a review protocol should be tracked and dated.** **The copyright for PRISMA-P (including checklist) is held by the PRISMA-P Group and is distributed under a Creative Commons Attribution Licence 4.0.**

From: Shamseer L, Moher D, Clarke M, Ghersi D, Liberati A, Petticrew M, Shekelle P, Stewart L, PRISMA-P Group. Preferred reporting items for systematic review and meta-analysis protocols (PRISMA-P) 2015: elaboration and explanation. BMJ. 2015 Jan 2;349(jan02 1):g7647.

For peer review only
